# A systematic review of the neural correlates of multisensory integration in schizophrenia

**DOI:** 10.1016/j.scog.2021.100219

**Published:** 2021-10-05

**Authors:** Cornelia Gröhn, Elin Norgren, Lars Eriksson

**Affiliations:** Department of Social and Psychological Studies, Karlstad University, Karlstad, Sweden

**Keywords:** Schizophrenia, Multisensory integration, fMRI, EEG, Neural correlates, Multimodal perception

## Abstract

Multisensory integration (MSI), in which sensory signals from different modalities are unified, is necessary for our comprehensive perception of and effective adaptation to the objects and events around us. However, individuals with schizophrenia suffer from impairments in MSI, which could explain typical symptoms like hallucination and reality distortion. Because the neural correlates of aberrant MSI in schizophrenia help us understand the physiognomy of this psychiatric disorder, we performed a systematic review of the current research on this subject. The literature search concerned investigated MSI in diagnosed schizophrenia patients compared to healthy controls using brain imaging. Seventeen of 317 identified studies were finally included. To assess risk of bias, the Newcastle-Ottawa quality assessment was used, and the review was written according to the Preferred Reporting Items for Systematic Reviews and Meta-analysis (PRISMA). The results indicated that multisensory processes in schizophrenia are associated with aberrant, mainly reduced, neural activity in several brain regions, as measured by event-related potentials, oscillations, activity and connectivity. The conclusion is that a fronto-temporal region, comprising the frontal inferior gyrus, middle temporal gyrus and superior temporal gyrus/sulcus, along with the fusiform gyrus and dorsal visual stream in the occipital-parietal lobe are possible key regions of deficient MSI in schizophrenia.

## 1 Introduction

We constantly encounter an abundance of sensory information that has to be successfully organized for us to be able to make sense of it. The converging processes of sensory modalities (e.g., auditory, visual, tactile modalities) required to generate a meaningful and coherent perception underlie the concept of multisensory integration (MSI; Talsma et al., 2010). For example, during a conversation with a friend in a busy restaurant, you will perceptually process your friend's voice (i.e., auditory stimuli) and articulations (i.e., visual stimuli) concurrently and rapidly to integrate them. This integration will increase your speech perception because MSI enhances perceptual acuity and improves detection, discrimination and response speed (Wallace et al., 2020). MSI does not only help us avoid cognitive overload and create meaning in the constant sensory information flood (Jensen et al., 2019), but also plays a crucial role for our daily functioning and well-being through guiding our responses to the complex outer world (Tseng et al., 2015).

Different types of research paradigms have been used to study the effects of MSI on behavior. Some focus on multisensory illusions that show how information from different sensory modalities can fuse together into one percept. One example is the *McGurk effect* in which a video of a person saying one phoneme (e.g., ‘ga’) is dubbed with a recording of another phoneme (e.g., ‘ba’) and resulting in the perceived illusion of a mixed phoneme (e.g., ‘da’) (McGurk & MacDonald, 1976). Other studies focus on the performance enhancement multisensory stimuli enable. One example is the redundant signals effect (RSE), which shows that responses are faster and more precise with stimuli presented in multiple sensory modalities compared to a single modality (Hershenson, 1962; Kinchla, 1974). Groundbreaking electrophysiology studies of neurons in the superior colliculus discovered important principles for MSI (Meredith and Stein, 1983, Meredith and Stein, 1986; Stein and Stanford, 2008). According to the principle of inverse effectiveness, multisensory enhancement is greater if the unisensory signals are of low intensity. In addition, multisensory facilitation is maximal when stimuli from different modalities are presented at the same time (temporal rule) at around the same place (spatial rule), and decreases with increased inter-stimulus onset (Stone et al., 2014).

### 1.1 Neural correlates of multisensory integration

Numerous brain-imaging studies of human and non-human animals (e.g., primates, rodents and cats) have provided evidence for MSI in, and anatomical pathways between, several different locations in the brain. These locations include both higher-order and lower-order cortical areas that are multisensory in nature. Important higher-order association areas are the temporal (i.e., superior temporal sulcus), parietal (i.e., intra parietal sulcus), and frontal (i.e., premotor cortex, prefrontal cortex) cortical regions (Cappe et al., 2012a; Ghazanfar and Schroeder, 2006). For example, the superior temporal sulcus (STS) contains neurons with multisensory properties (i.e., bimodal, trimodal or subthreshold neurons) that respond to stimuli in auditory, visual and somatosensory modalities (Baylis et al., 1987; Desimone and Gross, 1979), and has anatomical connections with visual and auditory cortical areas as well as the prefrontal cortex (Cappe et al., 2012a). Multiple neuroimaging studies on humans have shown that STS is responsive to multisensory stimuli, indicating that it possibly plays a general role in perception of speech and biological motion (see Calvert, 2001, for a review). Functional magnetic resonance imaging (fMRI) studies on humans indicate early interactions between lower-level sensory cortical areas in multisensory processing (see Cappe et al., 2012a, for a review), and the superior colliculus is an important subcortical site for multisensory integration (Meredith and Stein, 1983, Meredith and Stein, 1986; Stein and Stanford, 2008). Other subcortical structures like the striatum, cerebellum, amygdala and thalamus are also involved in multisensory processes (Engel et al., 2012).

However, the neural foundations of multisensory processes, constituting oscillations, networks and functional connectivity, are still not well-understood (Keil and Senkowski, 2018). The classical view suggests that integration occurs by convergence (Engel et al., 2012), and this means that integration is a feedforward and hierarchical process in which sensory information is first processed in primary sensory cortices and then integrated in higher order association areas and specialized subcortical regions (Meredith, 2002; Stein and Meredith, 1993). Integration by convergence cannot alone explain multisensory processing, in that, for example, we know cross-modal interactions take place already in primary cortices (Engel et al., 2012; Ghazanfar and Schroeder, 2006; Kayser and Logothetis, 2007). In their review, Keil and Senkowski (2018) present an integrative framework for the role of neural oscillations in multisensory integration built on recent studies. They propose that different frequency band-power (e.g., alpha-, beta-, gamma-, delta-, and theta-band) and functional connectivity networks are associated with different multisensory processes. For example, bottom-up processes, top-down processes and predictions across sensory modalities modulate integration, leaving MSI highly flexible and context-dependent. Nevertheless, our understanding of the neural correlates is still rather poor.

### 1.2 MSI and schizophrenia

Schizophrenia is a psychiatric condition characterized by both positive and negative symptoms. Hallucination, delusion and disorganization are referred to as positive symptoms while negative symptoms are a loss of premorbid functions such as loss of motivation and avolition (Bodén, 2016). The associated cognitive impairments in schizophrenia are hypothesized to result from atypical neural communication (van den Heuvel and Fornito, 2014), and several fMRI studies have identified altered anatomical neural connectivity in various brain regions (Allen et al., 2008; Crossley et al., 2016). A consequence of these neurocognitive alterations is the disability to acquire optimal adaptation for normal functioning with deficits in sensory integration (Heinrichs and Buchanan, 1988; Steinmann et al., 2019).

Many studies confirm that deficits in integration of sensory information in schizophrenia are apparent in visual, auditory and tactile modalities (e.g., de Gelder et al., 2003; Ferri et al., 2014; Vlcek et al., 2014). The impaired sensory perception is suggested to be related to passivity symptoms like hallucinations (Surguladze et al., 2001). The internal forward model is a widely accepted explanatory model of passivity symptoms in schizophrenia (Chen et al., 2015). It suggests that the characteristic passivity symptoms in schizophrenia derive from an inability to make accurate predictions about the perceptual or sensory outcome of their own intentional actions (Frith, 2005). Postmes et al. (2014) also argue that passivity symptoms in schizophrenia arise due to perceptual incoherence. They theorize that hallucination represents a coping mechanism to resolve incoherent multisensory experiences. Since sensory disturbances are apparent across several modalities and hallucinations are a multimodal phenomenon, Wallace et al. (2020) state that the understanding of these deficits will benefit from a multisensory perspective.

A growing number of studies have investigated MSI in schizophrenia with different paradigms and several of them present evidence of significant behavioral consequences of impaired audiovisual integration (e.g., de Jong et al., 2009; Ross et al., 2007; Stevenson et al., 2017). Facilitation effects on reaction times do not occur in schizophrenia patients in the same way as they do in healthy controls (Williams et al., 2010). In addition, patients experience the McGurk effect more rarely, which indicates less integration interference (Pearl et al., 2009). Findings manifest abnormalities in MSI with widened temporal binding window for individuals with schizophrenia (e.g., Foucher et al., 2007; Zhou et al., 2018), and impaired MSI appears to be most evident in speech-related audiovisual stimuli and worsened in a noisy environment (Wallace and Stevenson, 2014).

However, the results have not been consistent. The results of de Boer-Schellekens et al. (2014) indicate no deficits in MSI during tasks only engaging lower-level functions, supporting the notion that diminished sensitivity to visual temporal order inflicts on MSI rather than indicating deficits in MSI alone. de Gelder et al. (2003) found no difference in performance on simple MSI tasks between individuals with schizophrenia and healthy controls, but impairments in MSI were evident in schizophrenia during tasks involving speech. Williams et al. (2010) found impaired MSI using a basic audiovisual paradigm consisting of a simple detection task without any speech components. Still, some studies show no impairments in MSI (e.g., Aine et al., 2017).

In the meta-analysis of Tseng et al. (2015), it was concluded that impairments in MSI for non-emotional stimuli are evident in schizophrenia. Two studies reported increased multisensory facilitation effects with stimuli containing emotion-triggering aspects, for example a sad face or the sound of laughter (de Gelder et al., 2005; Van den Stock et al., 2011), while others report decreased integration of emotional stimuli (e.g., de Jong et al., 2009). Even though there are contradictory results, Tseng et al. (2015) lean towards suggesting impaired MSI for emotional stimuli as well.

### 1.3 Aberrant neural correlates in schizophrenia

In schizophrenia, there is a range of different brain regions that display abnormalities (Goghari et al., 2010; Shenton et al., 2001). When studied with magnetic resonance imaging (MRI), there is evidence for ventricular enlargement (e.g., DeLisi et al., 1997; Lieberman et al., 1996); peculiarities in medial temporal lobe structures including amygdala and hippocampus (e.g., Gur et al., 2007; Surguladze et al., 2006; Williams et al., 2007;); volume reduction in superior temporal gyrus (STG; e.g., Barta et al., 1990; Kim et al., 2003); parietal lobe atypicality (e.g., Arce et al., 2006); frontal lobe abnormalities (e.g., MacDonald et al., 2005; Taylor et al., 2007); and subcortical brain region deformity such as shape distortion in corpus callosum and volume abnormalities in basal ganglia (e.g., Ebdrup et al., 2010; Frumin et al., 2002; Gur et al., 1998). Additionally, deficits in the visual dorsal stream have been established repeatedly in the schizophrenia population (Koychev et al., 2011).

It has been proposed that it is relevant to look at these neurocognitive impairments from a perspective of larger scale neural circuits and cortical networks rather than just focus on local neural areas or specific brain regions (Onitsuka et al., 2013). New models are needed to explain the neurological variations in schizophrenia based on higher function, neural connectivity and brain rhythms rather than just anatomy and basic functions. One established model concerning this is the so-called dysconnectivity hypothesis that suggests abnormal interaction between different brain regions, especially a fragmentation of fronto-temporal connection, to be the reason for positive symptoms in schizophrenia (e.g., Friston et al., 2016).

Research on schizophrenia patients with electroencephalography (EEG) reveals aberrant oscillatory activity in several frequencies, including theta/delta bands (Boutros et al., 2008) and alpha/gamma bands (White et al., 2010). For instance, a decrease in synchronized gamma and beta power has been found when exposing the participants to different stimuli (Uhlhaas and Singer, 2013). When investigating neural oscillations in individuals with schizophrenia presented with auditory stimuli, several studies have found deficits in steady-state evoked potentials in gamma frequencies but also to some extent at lower frequency bands (e.g., Krishnan et al., 2009; Kwon et al., 1999). Deficits found in evoked oscillations when schizophrenia subjects processed visual information have been proposed to be a sign of reduced ability to arrange incoming sensory information accurately to oscillatory activity (e.g., Uhlhaas and Singer, 2010).

Kopell et al. (2011) showed that beta-receptor rhythms may mediate how healthy individuals integrate information from different sensory modalities. Together with the suggestion that these beta-receptor rhythms are weakened in the schizophrenic brain (Pittman-Polletta et al., 2015), a possible connection between oscillation alterations in schizophrenia and MSI is put forth. Furthermore, impairment in oscillations could possibly provide explanations for suggested problems with functional connectivity of cortical networks in schizophrenia (Uhlhaas and Singer, 2010).

### 1.4 Investigation rationale

MSI is associated with specific neural correlates, brain regions and oscillation rhythms (e.g., Cappe et al., 2012b; Keil and Senkowski, 2018). Contemporary behavioral studies indicate impairment in MSI for the schizophrenia population (Tseng et al., 2015), and multisensory impairments are evident in brain-imaging studies (e.g., Jardri et al., 2009; Silbersweig et al., 1995). It is relevant to continue to map disorder-related brain activity while processing multisensory stimuli in order to understand how the pathogenesis of schizophrenia hinders the afflicted from processing incoming sensory information in a coherent and cohesive way (Mäntylä et al., 2018). A greater understanding of how sensory processes are impacted in the brain of the schizophrenia population could be of use in treatment, and several studies suggest that new ways to help diagnose schizophrenia in the future are to be found in different types of brain imaging (e.g., Boutros et al., 2008; Kambeitz et al., 2015; Salvador et al., 2019). Because different brain-imaging techniques can shed further light on how MSI is expressed in schizophrenia, it is relevant to compile the existing literature in this field. It can also be considered that the most valuable studies are those directly comparing how the neural correlates during MSI differ between the population with schizophrenia and the healthy population. Accordingly, the aim of the present study was to perform a systematic review of this research with such direct comparisons of neural activity and connectivity. To our knowledge, there is no previous systematic review addressing this.

## 2 Method

### 2.1 Eligibility criteria

The target population was individuals diagnosed with schizophrenia or schizoaffective disorder (SP) according to the Diagnostic and Statistical Manual of Mental Disorders (DSM) or the International Statistical Classification of Diseases and Related Health Problems (ICD). The control group consisted of healthy individuals without current acute, severe or chronic disease. These healthy controls (HC) constitute the comparative reference on multisensory tests.

We selected a broad definition of multisensory tests based on MSI research to be able to include as many relevant articles as possible. If the study used an acceptable MSI paradigm, where two different sensory modalities were examined at the same time, it was included for further full-article assessment. All different sensory modalities were included (e.g., visual, auditory, olfactory, tactile, and taste). The outcome had to be measured with some type of brain-imaging technique (e.g., EEG, magnetoencephalography (MEG), fMRI).

Studies were included only if peer-reviewed, original articles in English, and accessible to the authors. Studies were excluded if they included a target population with mixed diagnoses, for example schizophrenia and/or schizoaffective disorder combined with other participants with bipolar or psychotic disorders.

### 2.2 Search strategy and selection process

**Fig. 1:**
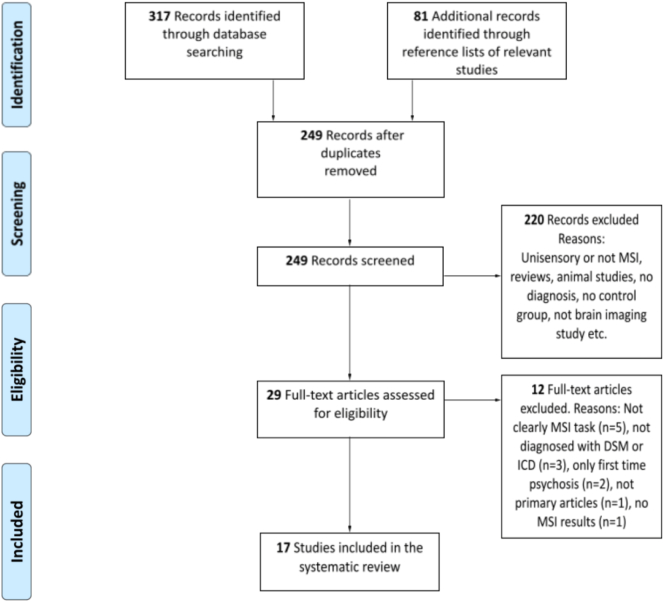
Flow of information through the different phases of the systematic review.

The review followed the Preferred Reporting Items for Systematic Reviews and Meta-analysis (PRISMA), and as suggested by PRISMA (Moher et al., 2009, Moher et al., 2015) the selection phase in this systematic review consisted of four different stages (Fig. 1). First, articles were identified through a database search. Second, the articles with duplicates removed were screened according to the selection criteria concerning title plus abstract. Third, the eligibility of each full-text article was rated according to predetermined criteria. Finally, approved articles were included in the systematic review.

The database search was performed in several different databases: PsychInfo, PubMed and Web of Science. Keywords from relevant articles and reviews were chosen as search terms. Search terms used were (“multisensory” OR “multi-sensory” OR “multimodal” OR “crossmodal”) AND (“integration”) AND (“neural” OR “cortical” OR “ERP” OR “fMRI” OR “EEG” OR “brain” OR “PET” OR “MEG” OR “oscillations”) AND (“schizophrenia” OR “schizo*” OR “schizoaffective” OR “psychosis”). Only peer-reviewed, original articles in English were included and records were searched from the earliest available until January 2020. In total, 317 records were identified through the database search. Two of the authors made the selection of articles, and disagreements were resolved by discussion between all three authors.

Identification was followed by screening in which duplicates were removed and titles plus abstracts were screened to check whether they met inclusion criteria. The reference lists from included articles were also searched and their relevant abstracts were screened for inclusion. Articles that did not meet the inclusion criteria were excluded (e.g., only unisensory or not clearly multisensory, no MSI task, systematic reviews and meta-analysis, animal studies, no diagnosis, no control group, did not measure outcome with brain-imaging techniques or inaccessible). Twenty-nine articles were assessed for eligibility. Two reviewers checked the eligibility of these full-text articles and inclusions of articles were based on consensus. Twelve additional articles were finally excluded. Three articles did not use DSM or ICD for diagnosis and two articles only included first time psychosis. Seven articles were excluded for several reasons, including the actual MSI task not studied with brain imaging (though other tasks were, *n* = 1); stimuli not presented at the same time and therefore not classified as MSI task (*n* = 2); primarily concerned other cognitive functions than MSI leading to difficulties in interpreting the results (*n* = 2); not a primary source (*n* = 1); and not describing any multisensory results (*n* = 1). Thus, 17 articles were included in the systematic review (see Fig. 1).

### 2.3 Quality assessment

The Newcastle-Ottawa Quality Assessment Scale (NOS; Wells et al., 2012) was used to assess the risk of bias in the results of the 17 included articles (see Appendix 1). The NOS comprises eight points or items grouped into three different sections, which are comparability, selection, and exposure. Each item was rated for a maximal score of one star and the maximal total score was eight. Like other systematic reviews, articles rated less than five out of eight were excluded (e.g., de Dieuleveult et al., 2017). The NOS was chosen since it is often used for systematic reviews and it is considered credible by the authors. Two authors performed the quality assessment of the selected studies, and there were no disagreements.

### 2.4 Data extraction and analysis of results

The collected studies were primarily grouped based on what brain-imaging technique had been used. Key characteristics in the data were described according to first author, title, publication year, number of participants, cognitive tests, participant characteristics, experiments, brain-imaging analysis, brain-imaging results, and performance when appropriate (see Appendix 2). Two authors made the data extraction and there were no disagreements.

For the analysis of results, the studies were grouped into social or non-social based on type of experiment and into fMRI and EEG/MEG based on brain-imaging technique. The results of how the neural correlates during MSI differ between the schizophrenia population and the healthy population were summarized for each brain-imaging technique. Since the experimental designs differed, a table (see Table 1) was created that presents the studies according to different conditions compared, brain-imaging results, performance results, unisensory processing, and brain regions.

**Table 1:** Overview of results. Articles are only presented once for each head category (exception brain regions), and articles that fit into more than one head category are sorted according to their primary results. Participants that are included in several studies (when performing different analysis of the same data) are only counted once in the table when equivalent results are reported.

Results overview		Number of articles		Number of participants (SP; HC)		Number of articles		Number of participants (SP; HC)	
		EEG (*n* = 10)	fMRI (*n* = 7)	EEG (*n* = 189; 191)	fMRI (*n* = 93; 93)	Non-social (*n* = 7)	Social (*n* = 10)	Non-social (*n* = 147; 147)	Social (*n* = 136; 137)
Conditions	Unisensory vs multisensory	3	2	65; 63	45; 45	4	1	94; 90	17; 18
	Different multisensory	3	2	53; 53	15; 15	0	5	0	68; 68
	A combination of the above	4	3	71; 75	33; 33	3	4	53; 57	51; 51
Results in neural activity	Sig. group differences	7	6	141; 146	76; 76	5	8	113; 117	104; 105
	No sig. group differences	2	1	48; 45	17; 17	1	2	33;30	32; 32
	Enhanced facilitation in SP	1	0	14; 15	0	1	0	14;15	0
Results in performance^b^	Sig. group differences	1	2	17; 17	33; 33	0	3	0	50; 50
	No sig. group differences	5	3	101; 99	33; 34	4	3	93;90	53; 54
	Enhanced facilitation in SP	2^a^	0	46; 57	0	2^a^	0	46;57	0
Unisensory processing	Deficits in SP	7	1	136; 138	17; 17	4	4	86; 87	67; 68
	Not reported	3	6	53; 53	76; 76	3	6	60;60	69; 69
Brain regions	Frontal/temporal	4	7	102; 113	93; 93	3	8	91; 102	104; 104
	Parietal/occipital	5	3	100; 104	60; 60	5	3	113; 116	47; 48

a Also found lower response accuracy in SP.

b Not all studies reported performance results.

## 3 Results

### 3.1 Quality assessment

The included studies all proved good quality according to the NOS (see Appendix 1). Ten studies had eight out of eight stars, four studies had seven stars and three studies had six stars. Thus, none of the studies had less than five stars and all 17 studies remained included.

### 3.2 Experimental design

The studies were sorted according to brain-imaging technique, fMRI (*n* = 7) and EEG (*n* = 10), and type of experiment, non-social (*n* = 7) and social (*n* = 10). The included studies compared unisensory to multisensory conditions (*n* = 5) or different multisensory conditions with one another (e.g., congruent vs incongruent, *n* = 5) or a combination of these (*n* = 7). All included studies used audiovisual stimuli only (see Table 1).

Experiments were considered non-social if they did not include a task concerning multisensory stimuli that consisted of speech, faces or other social stimuli (*n* = 7). Of the seven studies labeled as non-social, two used a simple multisensory detection task (Hanlon et al., 2016; Wynn et al., 2014). One study only included the instruction to “look and listen” during presentation of multisensory stimuli (Braus et al., 2002). Four studies involved audiovisual discrimination tasks (Balz et al., 2016; Sanfratello et al., 2018; Stone et al., 2011, Stone et al., 2014), two of which included manipulations of synchronization of audiovisual stimuli (Sanfratello et al., 2018; Stone et al., 2014). As mentioned in the introduction, it has been shown that visual deficits in SP are associated with deficits in the dorsal stream (i.e., “the where pathway” in the parietal lobe). By placing a multisensory stimulus in the peripheral view, which is the near condition in these experiments, the dorsal stream is engaged and the multisensory processing in the pathway can be recorded (Sanfratello et al., 2018; Stone et al., 2011, Stone et al., 2014). Sanfratello et al. (2018) and Stone et al. (2014) performed different analyses of the same dataset. In other words, they did not perform independent experiments but instead analyzed the same data.

Experiments were considered social if they included a task concerning social multisensory stimuli, like faces, voices or speech perception (*n* = 10). Three of the studies labeled as social used the McGurk paradigm or a version of it with some sort of discrimination task (Liu et al., 2016; Roa Romero et al., 2016a, Roa Romero et al., 2016b) or no specific task (Stekelenburg et al., 2013). One study had an emotional discrimination task based on face-voice stimuli (Müller et al., 2014). Two studies used an audiovisual speech perception paradigm including detection of semantic categories (Szycik et al., 2009, Szycik et al., 2013). Three studies investigated multisensory stimuli in the form of speech and gesture of which one used a content judgement task (Wroblewski et al., 2020) and two had a simple task for attentional purposes only (Straube et al., 2013, Straube et al., 2014). Szycik et al., 2009, Szycik et al., 2013 derive from the same dataset, as well as the two studies of Straube et al., 2013, Straube et al., 2014.

### 3.3 Participants

The total number of participants was evenly distributed between the group from the target population and the control group (SP: *n* = 282 and HC: *n* = 284).

Thirteen articles reported patient mean ages between 33.12 and 39.15 years (Balz et al., 2016; Hanlon et al., 2016; Müller et al., 2014; Roa Romero et al., 2016a, Roa Romero et al., 2016b; Sanfratello et al., 2018; Stekelenburg et al., 2013; Stone et al., 2014; Straube et al., 2013, Straube et al., 2014; Szycik et al., 2009, Szycik et al., 2013; Wroblewski et al., 2020). One article had a mean age of 25.1 (Braus et al., 2002) and three reported mean ages between 42.21 and 48.8 (Liu et al., 2016; Stone et al., 2011; Wynn et al., 2014). Thus, mean age in the included studies ranged from 25.1 to 48.8.

Five of the studies also included participants diagnosed with schizoaffective disorder (Hanlon et al., 2016; Sanfratello et al., 2018; Stekelenburg et al., 2013; Stone et al., 2014; Wroblewski et al., 2020). In three of these, the exact number of participants with schizoaffective disorder was not reported. However, in the two studies where the distribution is mentioned (Hanlon et al., 2016; Stekelenburg et al., 2013) the participants diagnosed with schizoaffective disorder are few in relation to the participants diagnosed with schizophrenia (SP-AFF: *n* = 3 and SP: *n* = 32).

Fifteen of the studies reported that patients were medicated with antipsychotic medication (Balz et al., 2016; Hanlon et al., 2016; Liu et al., 2016; Müller et al., 2014; Roa Romero et al., 2016a, Roa Romero et al., 2016b; Stekelenburg et al., 2013; Stone et al., 2011, Stone et al., 2014; Straube et al., 2013, Straube et al., 2014; Szycik et al., 2009, Szycik et al., 2013; Wroblewski et al., 2020; Wynn et al., 2014).

Eight studies reported mean duration of disorder between 8.24 and 14.33 years (Balz et al., 2016; Liu et al., 2016; Müller et al., 2014; Roa Romero et al., 2016a, Roa Romero et al., 2016b; Stekelenburg et al., 2013; Szycik et al., 2009, Szycik et al., 2013). One article focused on individuals with recent onset diagnosed schizophrenia, meaning they were first-episode, neuroleptic-naive and experienced their first psychiatric hospitalization during the experiment (Braus et al., 2002). Five studies specified that the participants were outpatients (Balz et al., 2016; Müller et al., 2014; Roa Romero et al., 2016a, Roa Romero et al., 2016b; Wynn et al., 2014).

### 3.4 Performance, response times and accuracy

Four studies reported multisensory performance deficits in SP due to lower response accuracy for SP than HC in some conditions (Roa Romero et al., 2016a; Stone et al., 2011) or across conditions (Stone et al., 2014; Wroblewski et al., 2020). Two studies reported longer RTs for SP than HC (Roa Romero et al., 2016a; Straube et al., 2013). Two articles reported greater multisensory facilitation in SP compared to HC when comparing visual unisensory trials with multisensory trials (Stone et al., 2011, Stone et al., 2014). Eight articles did not find any significant group differences or no indications of group differences in response times (Liu et al., 2016; Müller et al., 2014; Wynn et al., 2014), accuracy (Hanlon et al., 2016; Szycik et al., 2009, Szycik et al., 2013) or perceived illusions (Balz et al., 2016; Roa Romero et al., 2016b). Four articles did not report any performance data (Braus et al., 2002; Sanfratello et al., 2018; Stekelenburg et al., 2013; Straube et al., 2014).

### 3.5 Correlations with clinical measures

Several studies examined correlations between EEG, MEG or fMRI results and clinical measures. The clinical measures included Positive and Negative Syndrome Scale (PANSS), Scale for the Assessment of Negative Symptoms (SANS), Scale for the Assessment of Positive Symptoms (SAPS), Brief Psychiatric Rating Scale (BPRS), evaluation of cognitive domains with Measurement and Treatment Research to Improve Cognition in Schizophrenia Consensus Cognitive Battery (MATRICS), and medication dose. The outlines of these correlations are presented below. For PANSS, SANS, SAPS and BPRS a mixture of positive, negative and no correlations were found. Two studies found negative correlations, between positive symptoms and multisensory amplitude in the left intraparietal sulcus (IPS; Sanfratello et al., 2018) and between positive symptoms and connectivity to the left inferior frontal gyrus (IFG; Straube et al., 2014). In line with these, Müller et al. (2014) reported negative correlations between P100 amplitude and total BPRS scores. For all three, decreased amplitude or connectivity was associated with more severe symptoms. Wroblewski et al. (2020) found a negative correlation between middle temporal gyrus to superior temporal sulcus (MTG-STS) connectivity and the SANS attention subscale, indicating that decreased connectivity was associated with more severe symptoms. Furthermore, SANS avolition showed positive correlation with RT to auditory, visual and audiovisual stimuli, respectively, and negative correlation with hit rate (HR) to audiovisual stimuli. That is, more severe apathy was associated with longer RTs and lower HRs (Wynn et al., 2014). Several studies did not find any significant correlations with PANSS (Hanlon et al., 2016; Roa Romero et al., 2016a, Roa Romero et al., 2016b; Stone et al., 2011), PANSS Negative (Sanfratello et al., 2018; Straube et al., 2014) or BPRS (Braus et al., 2002; Stekelenburg et al., 2013; Wynn et al., 2014). (A number of non-significant correlations are left out here since it is out of scope for this systematic review.) Stone et al. (2014) reported that the relationship between gamma-band power and MATRICS scores was different for SP compared to HC. For example, correlations between multisensory gamma-band power and MATRICS scores were only found in HC.

Some of the included publications investigated the relationship between their results and medication dosage with the majority reporting no significant correlations (Balz et al., 2016; Liu et al., 2016; Müller et al., 2014; Roa Romero et al., 2016a; Sanfratello et al., 2018; Stekelenburg et al., 2013; Szycik et al., 2013). The exceptions are positive correlation between dosage and response rate in Stone et al. (2011), and positive correlation between dosage and gamma-band power in Stone et al. (2014).

### 3.6 EEG and MEG findings

Ten studies recorded neural activity with EEG and MEG, including various experimental designs and aims. Group differences between SP and HC in amplitudes and latencies were evident in a majority of studies. It was mainly reported that SP had reduced amplitudes and/or aberrant latencies compared to HC, indicating deficits in MSI (Balz et al., 2016; Sanfratello et al., 2018; Stone et al., 2011). One study found aberrant neural activity in early event-related potential (ERP) amplitudes and latencies, while late ERPs were only reduced to visual but not multisensory stimuli (Liu et al., 2016). Applying the principles of sub- and superadditivity (i.e., multisensory ERP minus the sum of unisensory ERPs), Stone et al. (2011) found enhanced facilitation in SP while two studies did not find any group differences (Liu et al., 2016; Wynn et al., 2014). Two studies found no group differences in global field power amplitude, a measure only reflecting effects simultaneously present in a large number of electrodes (Roa Romero et al., 2016a, Roa Romero et al., 2016b).

Oscillatory activity (i.e., theta, gamma and beta-band power) was found to be reduced in Balz et al. (2016) and Roa Romero et al. (2016a), and alpha-band power more strongly suppressed in Roa Romero et al. (2016b). In one study, both increases and decreases were found, mainly concerning greater gamma-band power in SP (Stone et al., 2014). However, this was due to failure in suppressing oscillatory power in all but one condition (i.e., gamma-band power was stronger than both baseline and HC in only one condition). This was the so-called near condition, where stimuli presented in the peripheral view were meant to activate the visual dorsal stream.

When comparing congruent to incongruent conditions of McGurk illusion trials or unisensory to multisensory trials, various interaction effects were found. SP had the opposite neural activity pattern compared to HC (Roa Romero et al., 2016b; Stekelenburg et al., 2013) or diminished differences between conditions (Liu et al., 2016; Roa Romero et al., 2016a). For example, HC displayed larger amplitudes in the congruent trials whereas SP displayed larger amplitudes in the incongruent trials, indicating that SP could not benefit from congruent multisensory information. Müller et al. (2014), on the other hand, only found reduced amplitudes for SP during emotional incongruent trials but no group differences during emotional congruent trials. This indicates that congruent sounds can affect responses and lead to neural responses similar to HC for emotional face-voice stimuli. However, the results from Müller et al. (2014) do not support the notion of SP having altered audiovisual integration since no difference was found between incongruent and congruent stimuli within the group.

Stekelenburg et al. (2013) focused on phonological predictions based on temporal and content information. The authors found that visual temporal information about sound onset did not have the suppression and/or speed-up effect on N100 for SP as it had for HC. These findings corresponded to group differences in multisensory integration. Roa Romero et al. (2016a) investigated crossmodal prediction error (PE) processing when recording theta-band oscillations and found interaction effects indicating that SP had impairments in resolution and in updating of violated predictions. These two studies indicate impairments in prediction processes during MSI in SP.

Seven out of ten articles reported deficits in unisensory processing (Liu et al., 2016; Müller et al., 2014; Wynn et al., 2014; Sanfratello et al., 2018; Stekelenburg et al., 2013; Stone et al., 2011, Stone et al., 2014), primarily in visual processing. Two of these did not find deficits in multisensory integration in SP (Müller et al., 2014; Stone et al., 2011).

Three studies investigated multisensory deficits in the visual dorsal stream, where two studies derive from the same dataset (Sanfratello et al., 2018; Stone et al., 2014). Results indicate deficits in the visual dorsal stream during multisensory processing in SP (Sanfratello et al., 2018; Stone et al., 2014). In line with these results, Liu et al. (2016) reported reduced amplitudes in response to face stimuli in multisensory conditions, indicating that impairments in face processing are also evident in face-voice integration.

Five studies located group differences in multisensory processing in parietal and occipital regions (Balz et al., 2016; Liu et al., 2016; Müller et al., 2014; Sanfratello et al., 2018; Stone et al., 2011), and four studies located differences in frontal/temporal regions (Roa Romero et al., 2016a, Roa Romero et al., 2016b; Stekelenburg et al., 2013; Stone et al., 2014). Source estimations using a linear distributed inverse solution based on a Local Auto-Regressive Average (LAURA) in Stekelenburg et al. (2013) revealed several abnormalities (i.e., under activation or displaced activation) in neural networks of audiovisual integration. These networks involved auditory cortex, superior temporal gyrus, middle temporal gyrus, and inferior frontal gyrus.

### 3.7 fMRI findings

There are some findings of subcortical abnormalities (Braus et al., 2002; Szycik et al., 2009) and alterations in cortical areas were discovered in all of the fMRI-studies. The neural abnormalities in the cortical areas were predominantly focused around the STS (Straube et al., 2013, Straube et al., 2014; Szycik et al., 2013; Wroblewski et al., 2020); the STG (Hanlon et al., 2016; Szycik et al., 2009); the IFG (Straube et al., 2013, Straube et al., 2014; Szycik et al., 2009, Szycik et al., 2013; Wroblewski et al., 2020); the fusiform gyrus (FG; Szycik et al., 2009; Wroblewski et al., 2020); and the visual dorsal stream (Braus et al., 2002; Hanlon et al., 2016). Two articles found reduced connectivity in both STS and IFG for SP during audiovisual trials (Straube et al., 2014; Szycik et al., 2013), whereas one found reduced connectivity only in the MTG/STS region (Wroblewski et al., 2020) and another suggested dysfunction only in the left IFG (Straube et al., 2013). Yet, it is important to add that the decreased connectivity in the left IFG was mainly found in incongruent trials in Szycik et al. (2013), a condition that was not part of the experiment made by Wroblewski et al. (2020). This could possibly be connected to the results with dysfunctions in abstract, but not concrete, multisensory information processing found in STS, bilateral IFG (Straube et al., 2014), and the left IFG (Straube et al., 2013).

Overall, there are tendencies of lower responsiveness and less activation in the SP group (Braus et al., 2002; Hanlon et al., 2016; Straube et al., 2013, Straube et al., 2014; Szycik et al., 2009, Szycik et al., 2013). In three studies, however, the impairments are not as distinct as expected, with many similarities between HC and SP (Straube et al., 2013, Straube et al., 2014; Wroblewski et al., 2020).

Two articles report that impairments in audiovisual integration originate from dysconnectivity in both the STS and the IFG, especially when SP is exposed to incongruent stimuli (Szycik et al., 2013) or when integrating stimuli with abstract content (Straube et al., 2014). Moreover, abnormal MSI processing is explained to derive from deficits in inhibition (Hanlon et al., 2016); impairments in the verbal pathway (Wroblewski et al., 2020); defected speech motor system in the right hemisphere and decreased lateralization of speech functions to the left hemisphere (Szycik et al., 2009); alterations in cortical as well as subcortical activation patterns, mainly focused around high-order frontoparietal cortex and thalamus (Braus et al., 2002); and a failure to activate the left IFG and posterior MTG (Straube et al., 2013). In addition, Straube et al. (2014) suggest that dysconnectivity of left STS and prefrontal cortex could be associated with failure to utilize the hemispheric functions adequately in a context-dependent manner.

### 3.8 Main findings

The main findings are summarized and presented in Table 2. In short, reduced neural activity and additional aberrant neural response-patterns were evident in SP during MSI tasks in both fMRI and EEG/MEG studies, and distributed across several brain regions and networks. Furthermore, no difference in outcome was ascertained between social and non-social tasks (see Table 1).

**Table 2:** Main findings in brain-imaging results showing only results reflecting group differences and/or interaction effects in activity, connectivity, oscillations, amplitudes and latencies during multisensory-task recordings. The number of studies is indicated by *n*.

	fMRI	EEG
Reduced activity	*Brain regions with reduced activity/absence of activation*: Dorsolateral prefrontal cortex (*n* = 1), thalamus (*n* = 1), STG (*n* = 1), MTG (*n* = 1), STS (*n* = 1), and IFG (*n* = 1) and overall weaker activation (*n* = 1) *Networks with reduced activity/absent of activation*: Dorsal visual stream (*n* = 1), default mode circuit (*n* = 1) *Reduced connectivity*: STS-MTG (*n* = 1), STS in general (*n* = 2), IFG in general (*n* = 2)	*Oscillations*: Reduced frontal theta-band (*n* = 1) and occipital gamma-band and beta-band power (*n* = 1) *Amplitudes*: Reduced early amplitudes in parietal/occipital regions (*n* = 3) Reduced amplitude in early evoked potentials in temporal-occipital and parietal occipital regions (*n* = 1) Reduced amplitudes in parietal (i.e., IPS SPG and IPG) and temporal (i.e., STS and STG) regions (*n* = 1)
Aberrant activity	*Contrasting patterns to HC between conditions*^a^: In cuneus (*n* = 1), insula (*n* = 1) cingulate gyrus (*n* = 2), STG (*n* = 1), STS (*n* = 1), hippocampus (*n* = 1), precuneus (*n* = 1), caudate nucleus (*n* = 1), FG (*n* = 1), middle occipital gyrus (*n* = 1), parahippocampal gyrus (*n* = 1), transverse temporal gyrus (*n* = 1), MTG (*n* = 2), and IFG (*n* = 2) *Brain regions with other aberrant activation*: Thalamus (*n* = 1), precuneus (*n* = 1, MTG (*n* = 1), STS (*n* = 1), STG (*n* = 1), FG (*n* = 2), nucleus accumbens (*n* = 1) *Aberrant connectivity:* STS-IFG (*n* = 1) STS-frontal cortex (*n* = 1) *Contrasting patterns to HC between conditions*^a^: STS-connectivity in general (*n* = 1) IFG-paracentral lobule connectivity (*n* = 1) STS-precuneus/cuneus connectivity (*n* = 1) Diminished IFG-connectivity differences between conditions^a^ (*n* = 1)	*Contrasting patterns to HC between conditions*^a^: Alpha-band power more strongly suppressed in auditory and/or frontal areas (*n* = 1) Stronger power and failure to suppress frontal gamma-band power (*n* = 1) Delayed peak latency in parietal regions (*n* = 2) *Diminished differences between conditions*^a^: In frontal theta-band power (*n* = 1) In fronto-central, medio-central and parietal amplitudes (*n* = 3) Under or displaced activation in auditory cortex, STG, MTG and IFG (*n* = 1)

a Unisensory vs multisensory, incongruent vs congruent, congruent vs illusory.

Summary of reduced activity in neural correlates during MSI tasks:

• Reduced amplitudes in occipital/parietal ERPs during MSI tasks were revealed when using EEG or MEG (Balz et al., 2016; Liu et al., 2016; Sanfratello et al., 2018; Stone et al., 2011).
• Reduced oscillatory activity was found in gamma/beta-band power in occipital/parietal regions (Balz et al., 2016); in theta-band power in frontal regions (Roa Romero et al., 2016a); and stronger suppression in alpha-band oscillations was observed (Roa Romero et al., 2016b).
• Reduced fMRI-activation was seen both as an overall pattern (Szycik et al., 2009) and in specific brain regions (Braus et al., 2002; Hanlon et al., 2016), for example bilaterally in secondary auditory regions, the default-mode network and the dorsal visual pathway, and in the right thalamus and prefrontal cortex. In addition, reduced activation was visible during MSI tasks with an abstract content (Straube et al., 2013).
• Reduced connectivity was found in all conditions between STS and frontal cortex (Straube et al., 2014), as well as for the STS and MTG (Wroblewski et al., 2020), and during congruent stimuli for left IFG to a range of different brain regions (Szycik et al., 2013).

Summary of aberrant neural activity during MSI tasks:

• Aberrant neural processes (i.e., amplitudes, oscillations, activity and connectivity) were made evident by various interaction effects where SP often demonstrated contrasting response patterns compared to HC (Müller et al., 2014; Roa Romero et al., 2016a, Roa Romero et al., 2016b; Stekelenburg et al., 2013; Szycik et al., 2009, Szycik et al., 2013).
• In SP, differences between conditions (congruent vs incongruent or illusory) were either diminished (e.g., Stekelenburg et al., 2013) or SP had the opposite neural activity pattern to HC (e.g., Roa Romero et al., 2016a, Roa Romero et al., 2016b; Szycik et al., 2009, Szycik et al., 2013), demonstrating aberrant processing of MSI stimuli.
• As mentioned above, SP mainly demonstrated reduced neural responses to conditions meant to elicit multisensory integration processes (e.g., Roa Romero et al., 2016a, Roa Romero et al., 2016b; Stekelenburg et al., 2013; Szycik et al., 2009, Szycik et al., 2013). However, in some studies SP only had deficits in the incongruent (Müller et al., 2014) or unisensory (Stone et al., 2011) conditions, indicating normal integration or even enhanced multisensory facilitation.
• This additional aberrant neural activity was associated with both parietal/occipital regions (Liu et al., 2016; Müller et al., 2014) and fronto-temporal regions (Roa Romero et al., 2016a, Roa Romero et al., 2016b; Stekelenburg et al., 2013; Stone et al., 2011). More specifically, it involved the posterior STS (Szycik et al., 2013), left posterior STS (Straube et al., 2014), the FG (Szycik et al., 2009; Wroblewski et al., 2020), and auditory cortex, STG, MTG and IFG (e.g., Stekelenburg et al., 2013).

## 4 Discussion

The research on MSI in schizophrenia recorded with EEG, MEG and fMRI is still a relatively unexplored, but growing, research topic. In this review, seventeen studies were included and their results lead us to two main insights. First, the activity in neural correlates during MSI is aberrant and mainly reduced in SP compared to HC. Second, these differences are distributed across several brain regions and networks associated with sensory and multisensory processes. However, we could not find any clear connections between experimental design, brain-imagining technique and results in neural activity, making it difficult to draw any further conclusions about how neural and performance outcomes may be modified by experimental design and brain-imaging technique.

### 4.1 Aberrant and reduced neural responses in SP

In comparing SP to HC, thirteen articles reported significantly reduced neural responses in SP whereas only three reported no differences and one enhanced facilitation in SP. The differences were either due to reduced activity, connectivity, oscillations and ERPs and/or various interaction effects in a majority of the studies. These results are mostly interpreted as altered audiovisual integration at the neural level in SP, which might reflect deficits or anomalies in MSI on performance level (e.g., Balz et al., 2016; Roa Romero et al., 2016b; Sanfratello et al., 2018; Szycik et al., 2009). It is also suggested that SP lack the facilitating or enhancement effect of congruent multisensory information seen in HC (e.g., Roa Romero et al., 2016a, Roa Romero et al., 2016b; Stekelenburg et al., 2013; Szycik et al., 2009, Szycik et al., 2013). This means that healthy individuals have different neural responses to congruent compared to incongruent audiovisual information, which is helpful when interpreting and responding to the outside world. The lack of difference in neural response in SP might indicate difficulties both in integrating congruent stimuli and in differentiating between incongruent stimuli. Consequently, the perceptual benefits of MSI are impaired for SP. Aberrant neural activity has been associated with performance deficits in MSI in previous research (Magnée et al., 2009; Sass et al., 2013; for reviews see also Keil and Senkowski, 2018, Tseng et al., 2015, and Wallace et al., 2020). The inability to respond appropriately to audiovisual information could be due to impairments in prediction processing, such as deficits in making predictions about sounds from visual information (Stekelenburg et al., 2013), and/or impairments in prediction error processing (Roa Romero et al., 2016a). This is in line with the integrative framework that Keil and Senkowski (2018) present, in which they theorize that SP have deficits in generating intersensory predictions and evaluating stimulus congruence as well as deficits in the resolution of incongruence. The internal forward model system illustrates how impairments in prediction error processing can cause positive symptoms like hallucinations in SP. In healthy individuals, because of sensory prediction the response to the sound of their own voice is attenuated in auditory cortex when speaking. This attenuation is not evident in SP, especially for those who report auditory hallucinations (Frith, 2005).

However, the dataset in this review points in several directions and some studies did not find any group differences in neural activity. Stone et al. (2011) even found that multisensory facilitation was enhanced in the SP group. In Liu et al. (2016) aberrant neural activity was found only in early ERPs while late ERPs were only reduced to visual but not multisensory stimuli. Müller et al. (2014) compared the emotional rating of congruent to incongruent face-voice stimuli and found deficits only in the incongruent condition. They concluded that their findings demonstrate that early ERPs (i.e., P100 amplitude) can be affected by congruent sound, but only when the stimuli have emotional valence and not when neutral. Furthermore, Wynn et al. (2014) did not find any difference between groups in ERPs, and Straube et al., 2013, Straube et al., 2014 and Wroblewski et al., 2020 found that SP did not differ from HC in brain activity or connectivity during MSI tasks involving co-verbal gestures with concrete content. Taken together, the studies suggest aberrant and reduced neural activity reflecting deficits in multisensory integration. However, at least under some circumstances, SP seem to have intact MSI with the ability to benefit from audiovisual cues, and maybe even compensate for deficits in visual processing during congruent multisensory stimuli.

### 4.2 Brain regions and networks associated with deficits in MSI in SP

There are multiple brain regions, both cortical and subcortical, found to be impaired in the different articles included in this review. Our results, however, indicate that the brain regions with aberrant processing during MSI in SP are the STS, the STG, the FG, the MTG and the IFG. Moreover, deficits were seen in the visual dorsal stream, with impairments mainly visible in the IFG and the STS. This is comparable to previous findings, as aberrant processing in SP has been found previously in the IFG (Arce et al., 2006), the MTG (Surguladze et al., 2006), the FG (Onitsuka et al., 2003) and the STG (Barta et al., 1990). The STS is not frequently reported as a region normally associated with the neural deficits of the SP population. However, there are studies showing a dysfunctional pattern in most parts of the brain, and describing the deficits in larger regions (i.e., temporal lobe/cortex) makes it harder to distinguish if the STS is affected or not (Shenton et al., 2001). On the other hand, the STS is a region that has been found to be very important for MSI processing (Desimone and Gross, 1979). There is a possibility that the STS function is mainly reduced in SP when responding to MSI stimuli, which could explain why this is not a region typically found to be impaired.

There are indications that the dorsal visual stream is associated with dysfunction in SP during MSI in the present review (e.g., Sanfratello et al., 2018). Reduced activity in the extrastriate visual cortex, which is part of the dorsal visual stream, has previously been seen in the SP population during perceptual organization of visual information (Silverstein and Keane, 2011). Other articles also indicate deficits in the dorsal visual stream for SP (e.g., Butler and Javitt, 2005), seemingly involved during MSI (Kaposvari et al., 2015). This could suggest that these dysfunctions are due to aberrant unisensory processing affecting MSI rather than aberrant multisensory processing in itself. However, in Stone et al. (2014), deficits were seen in the dorsal visual stream for unisensory visual stimuli besides the multisensory deficits, and the authors analyzed whether the unisensory deficits could be directly mapped onto the changes in MSI response. They could not, which indicates that the aberrant processing in the visual dorsal stream during MSI does not emerge directly from deficits in unisensory processing.

In some articles in this systematic review the impairments are located in particular in the left IFG (e.g., Szycik et al., 2013), a region also known as Broca's area. One study finds deficits only in the left STS (Straube et al., 2014), which is part of Wernicke's area. Both Broca's area and Wernicke's area are strongly associated with speech functions and language ability (Kolb and Whishaw, 2015). The articles in our systematic review showing deficits in these areas all use social multisensory stimuli accompanied with speech. Furthermore, as mentioned in the introduction, the STS bilaterally is thought to be involved in social functions such as both speech perception and biological motion (Calvert, 2001). This agrees with our findings, since the disturbances in the STS are only visible in the studies using gestures or lip movements as part of the multisensory stimuli. This could indicate that these deficits are more strongly associated with speech perception or language comprehension, or perhaps social visual information, rather than multisensory processing. On the other hand, also mentioned in the introduction, the STS is an important region for multisensory processing. In addition, the left IFG, together with its right homolog, has proved to be of importance specifically for audiovisual integration of speech in healthy individuals (Curcic-Blake et al., 2013; Ojanen et al., 2005; Pekkola et al., 2005). This makes it reasonable to believe that our results do indicate reduced MSI.

Several of the articles find deficits in the IFG bilaterally (e.g., Szycik et al., 2009). The right IFG also takes a great part in functions involving attention and inhibition (Hampshire et al., 2010), and both attention and inhibition are known to be impaired in SP (e.g., Arce et al., 2006; Nuechterlein et al., 2015). However, when using experiments where the paradigm is designed to test automatic audiovisual integration processes, not dependent on attention, deficits are still visible for SP (de Gelder et al., 2003). Regarding inhibition, Hampshire et al. (2010) argue that the right IFG is more likely to be part of a network that tunes in on task-relevant stimuli rather than alone responsible for inhibitory control. They further conclude that the right IFG has a far greater role than just inhibition. Our systematic review opens up to the possibility that this greater role could involve multisensory processing.

Three areas additionally observed to be involved in MSI are the FG, the MTG and the STG (Pehrs et al., 2014; Surguladze et al., 2006), which all have been indicated to be impaired in this systematic review. All of these are also found to be associated with proneness to hallucinations and the production of them (Kim et al., 2003; Kunzelmann et al., 2019; Zhang et al., 2017). As previously mentioned, the link between multisensory disturbances and symptoms like hallucinations or distorted reality is commonplace (Frith, 2005). The positive symptoms in schizophrenia are often explained with the dysconnectivity hypothesis (e.g., Friston et al., 2016), and dysconnectivity was found in several of the articles. They included dysconnectivity both in and between multiple brain regions throughout all of the lobes, including dysconnectivity in the parietal cortex. Moreover, decreased parietal connectivity has been demonstrated to lead to decreased MSI facilitation (Brang et al., 2013). Possibly, there is a link between the dysconnectivity typical of the positive symptoms in SP and the evident dysconnectivity seen with decreases in multisensory facilitation. The correlations with clinical measures found in this systematic review further indicate this link as positive symptoms were negatively correlated to MSI amplitudes in one article (Sanfratello et al., 2018) and to connectivity in IFG in another (Straube et al., 2014). Furthermore, reduced neural activity was associated with more severe symptoms in several studies (e.g., Müller et al., 2014; Wroblewski et al., 2020).

### 4.3 Strengths and limitations

This systematic review followed the PRISMA guidelines in the aspiration to ensure a fair and reliable selection process and to improve the quality of the systematic review (Moher et al., 2009, Moher et al., 2015). The literature search was made in the three databases PubMed, PsycInfo and Web of Science with multiple search terms and a broad definition of MSI, and all possible brain-imaging techniques were included. Furthermore, all included articles had to meet pre-determined quality standards according to the NOS (Wells et al., 2012). Two reviewers checked the eligibility of the 29 full-text articles that emerged from the first screening, and the ultimate inclusion of 17 articles was based on consensus. Even though these measures were taken to find as many relevant articles as possible, some articles investigating MSI in schizophrenia with brain-imaging techniques might not be included. This could be due to search limitations such as time constraints, overlooked relevant search terms, and/or articles published after the search period. It could also be due to researchers not labeling their experiments as MSI-related even though they might be. It is possible that the unintended exclusion of relevant search terms could have affected our findings. One possible way to address this in future reviews could be to add search terms like “sensory”, “illusion” and “speech” so that articles not labeled as MSI-related could be included in the initial search process. The definition of MSI chosen by the authors, also affecting the inclusion process, could be categorized as too wide or too narrow compared to other definitions. Since the identification of articles in the three databases was divided between the two reviewers, there is a risk of bias in the selection process. If the reviewers had performed the identification and screening of articles in all three databases individually to make sure the same articles were included for assessment of eligibility, it would have decreased the risk of bias and the risk of missing relevant articles.

Since this review focuses on an emerging research field, it is based on a rather limited sample with 17 included articles. Furthermore, because the dataset is the same for some of the articles the number of included participants is even smaller than what perhaps could be expected (SP: *n* = 282; HC: *n* = 284). There are no optimal numbers of included articles in a systematic review and the number of relevant articles is highly dependent on the size of the research field. However, with 17 included studies the risk of overlooking relevant articles is that just one article could affect the results and conclusions of the systematic review, whether they point in the same direction or not. Furthermore, a limited number of participants increase the risk of making a type II error (i.e., not finding a difference that would be visible in a larger sample). This risk is even greater with greater variance in group characteristics. Even though all groups from the included articles were evaluated within the NOS (Wells et al., 2012), and considered equal, there is still a possibility for some variance. For example, one study included neuroleptic-naïve first-episode schizophrenic patients while others included patients with illness durations up to 16 years.

A difficulty in this systematic review has been to compile and interpret the included studies that are quite heterogeneous. The experimental designs differ greatly due to different focuses in aim. For example, some of the studies focus on abstract thinking ability rather than MSI per se, some compare unisensory to multisensory conditions and others incongruent versus congruent. Therefore, there is a possibility that the aberrant neural activity is better explained by differences in experimental design, for example complexity of the task or if the task was social or non-social. However, we could not find any connections between results in neural activity and type of task. In our results, both social tasks and non-social tasks were associated with differences in neural processing between SP and HC. This further strengthens the assumption that MSI processing is aberrant in SP, since it cannot be explained with the tasks having social elements. Still, it is possible that there are connections in more detailed measures between different tasks and activity or connectivity, but that this is not evident in the present review. Furthermore, it could be that the study sample is too small and too versatile to find underlying experimental factors behind the outcomes. There is a possibility that these differences will become evident when more research is available. For example, underlying factors could be how MSI is defined and computed, differences in stimulus characteristics and/or analytic methods, and how conditions are compared.

A further limitation is that schizophrenia is a heterogeneous disorder (Mohr et al., 2004). For example, not all individuals diagnosed with schizophrenia show catatonic behavior, even though it is a symptom of the disease. Therefore, it is a possibility that the inconsistencies showing in this systematic review are a consequence of that heterogeneity. It could be that not all individuals with schizophrenia have impairments in MSI, at least not to the same extent. Furthermore, some of the individuals with schizophrenia could have found ways to compensate. Due to the differences in design, we cannot draw any conclusions concerning under what specific conditions MSI is affected in schizophrenia or its behavioral manifestation. However, the combined experimental designs in these studies mirror how people come across MSI in their everyday life, increasing external validity and the possibility to generalize results. The variety in group characteristics and the range of different types of design are both representative of reality, clarifying that the differences we can see from this systematic review are evident for several types of conditions and across groups.

The aim of this systematic review was to compile and analyze evidence of MSI impairments in schizophrenia using performance measures. However, although aberrant activity was a prominent finding in this review, it was not clearly reflected in the performance measures. Five out of the thirteen articles that reported performance measures found impairments in response accuracy, RTs or both (Roa Romero et al., 2016a; Stone et al., 2011, Stone et al., 2014; Straube et al., 2013; Wroblewski et al., 2020). In six studies, differences in neural activity were found, but no difference in RTs, accuracy or perceived illusions (Balz et al., 2016; Hanlon et al., 2016; Liu et al., 2016; Müller et al., 2014; Roa Romero et al., 2016b; Szycik et al., 2009, Szycik et al., 2013). (Note that Szycik et al., 2013, report the same performance results as Szycik et al., 2009, and that these two are counted here as a single study.) Even though behavioral performance was not the focus of this review, the authors believe it is important to reflect upon this result. It is possible to argue that it affects the validity and importance of the findings negatively. What conclusions can we draw from differences in neural activity between SP and HC when these are not reflected in expected behavioral differences?

In this systematic review, all different sensory modalities were included in the search process, but only audiovisual studies were included. This indicates that there is a majority of studies on neural correlates during audiovisual integration, and that the integration of other senses might be somewhat neglected in the empirical research. As mentioned in the introduction, it is well known that schizophrenia is associated with impairments in several sensory modalities. To understand the impairments in MSI fully, the associated neural correlates and clinical consequences, it is important to investigate other sensory modalities (e.g., tactile, olfactory, taste). Additionally, it is important to investigate impairments in multisensory integration of bodily signals, somatosensory input, especially since somatosensory impairments have been theorized to explain the “self-disorders” in schizophrenia (Postmes et al., 2014). Self-disorder is the difficulty to distinguish between self and other, which may cause passivity symptoms characteristic of schizophrenia (Chen et al., 2015). Multisensory impairments probably affect both environmental and bodily sensory signals in schizophrenia.

### 4.4 Conclusions and further directions

This is the first systematic review examining how the neural correlates during multisensory integration differ between individuals with schizophrenia and the healthy population. We found aberrant and reduced neural activity measured with EEG, MEG and fMRI, presumably reflecting deficits in multisensory integration in schizophrenia. This was evident in several brain regions involving multisensory integration, mainly in the temporal cortex (i.e., STS/STG, MTG and FG) as well as in frontal (i.e., IFG) and occipito-parietal (i.e., the dorsal stream) regions. However, results indicate that MSI could be intact during some conditions. Furthermore, difference in neural activity was not always reflected in performance deficits.

Further research could investigate the possibility that MSI is reduced in schizophrenia patients only during some conditions to find out the underlying mechanism(s) triggered by those specific conditions. Since this systematic review did not see any clear differences in neural correlates in schizophrenia patients when performing an audiovisual task categorized as social instead of non-social, it would be interesting to investigate this further. Moreover, clarifying the role of other sensory modalities like the tactile modality, and the integration of somatosensory input in schizophrenic self-disorders seem justified. The relationship between deficits in unisensory and multisensory processing is unclear and future research ought to continue to determine how they are related. If some individuals are able to compensate for deficits, it would be beneficial for others who suffer impairments in MSI if future research would investigate this topic further.

## CRediT authorship contribution statement

**Cornelia Gröhn**: Conceptualization, Formal analysis, Investigation, Writing – Original Draft.

**Elin Norgren**: Conceptualization, Formal analysis, Investigation, Writing – Original Draft.

**Lars Eriksson**: Conceptualization, Methodology, Writing – Review & Editing, Supervision.

## Declaration of competing interest

The authors declare that they have no known competing financial interests or personal relationships that could have appeared to influence the work reported in this paper.

## Figures and Tables

**Table:** 

	Quality assessment criteria								
	Selection (4 stars)				Comparability (2 stars)		Exposure (2 stars)		
	Is the population definition adequate?	Representativeness of the population	Selection of controls	Definition of controls	Study controls for schizophrenia	Study controls for additional factor	Same method of ascertainment for cases and controls	Non-response rate	
Study	Schizophrenia or schizoaffective disorder according to DSM or ICD	Representative of the schizophrenia population	Same community	Healthy individuals with no current or chronic major disease	Diagnosed schizophrenia compared to healthy controls	Gender, age, education/cognitive functioning (e.g., IQ)	Methods are identical	Same rate for both groups	Quality score (max. 8 stars)
Balz et al., 2016	*	*	*	*	*	*	*	*	8
Braus et al., 2002	*	–	*	*	*	*	*	*	7
Hanlon et al., 2016	*	*	*	*	*	*	*	*	8
Liu et al., 2016	*	*	*	*	*	–	*	*	7
Müller et al., 2014	*	*	*	*	*	*	*	*	8
Roa Romero et al., 2016a	*	*	*	*	*	*	*	*	8
Roa Romero et al., 2016b	*	*	*	*	*	*	*	*	8
Sanfratello et al., 2018	*	*	*	*	*	*	*	*	8
Stekelenburg et al., 2013	*	*	*	*	*	*	*	*	8
Stone et al., 2011	*	*	*	*	*	*	*	*	8
Stone et al., 2014	*	*	*	–	*	–	*	*	6
Straube et al., 2013	*	*	–	*	*	–	*	*	6
Straube et al., 2014	*	*	–	*	*	–	*	*	6
Szycik et al., 2009	*	*	*	*	*	–	*	*	7
Szycik et al., 2009	*	*	*	*	*	–	*	*	7
Wroblewski et al., 2020	*	*	*	*	*	*	*	*	8
Wynn et al., 2014	*	*	*	*	*	*	*	*	8

**Table:** 

Title, first author (year of publication)	Study characteristics								Brain imaging analysis	Main results and major conclusions
	Participants (number; female/male; age interval; mean (SD))		Clinical and cognitive assessment (Test; mean total score)		Controlled/matched for	Experiment				
	Schizophrenia/Schizoaffective	Healthy controls	Subjects	Control group		Tasks and instructions	Conditions	Brain imaging technique		
Balz et al. (2016)	15; 3/12; -; 33.87 (7.23)	15; 3/12; -; 36.13 (7.91)	DSM-IV-TR, PANSS; 74.07 (8.81), BACS; 248.27 (41.65) Illness duration, 9 (4.8) Antipsychotic medication, 15	SCID non-patient edition, BACS; 259.60 (36.89)	Age, gender, education, handedness, normal hearing, normal or corrected-normal vision, no neurological disorders, alcohol or substance abuse. 40% underwent a multi-drug screening, all negative results.	Sound-induced flash illusion (SIFI) Instructions: Report the number of perceived visual stimuli by pressing a button with the index, middle, or ring finger of their right hand to indicate whether they perceived 0, 1, or 2 flashes, respectively.	1) A0V1 2) A0V2 3) A1V1 4) A2V0 5) A2V1 6) A2V2 Numbers denote the number of auditory (A) and visual (V) inputs. A = tone. V = flash.	EEG; ERP	“ERPs were analyzed in a time interval from 0 to 400 ms following stimulus onset. Non-parametric tests, separately between the different percepts, between groups, and for the Perception by Group interaction. To test differences between the processing of multisensory and unisensory stimuli, visual trials, as well as auditory trials were analyzed. Non-parametric tests between conditions, between groups and for the Condition by Group interaction. To test if there are differences between the processing of multisensory and unisensory stimuli, an analogous analysis was carried out for unisensory visual stimuli with the within-subject factor Condition and the between-subjects factor Group.”	“Analysis of ERP data revealed altered multisensory integration effects in SCZ compared to HC. The study complements recent reports of altered multisensory integration in the McGurk illusion, suggesting processing differences between SCZ and HC in various prominent multisensory illusion paradigms.”
Braus et al. (2002)	12; 6/6; -; 25.1 (4.8); school education, 10.6 ± 1.8 years,	11; 5/6; -; 29.4 (6.2); school education, 12.0 ± 1.4 years.	DSM-IV; ICD-10; BPRS 49.9 (5.7). First psychiatric hospitalization.	–	Age, gender, education, handedness, no substance abuse. For HC: no history of significant medical or psychiatric illnesses For SP: No history of neurologic disorders, no abnormalities showing in neurologic examination. None of the participants had ever received neuroleptic medication before being enrolled in the study. All patients were neuroleptic-naive, having their first schizophrenic episode with the predominance of delusions, hallucinations, and distrust. Urine screening for drug usage was negative. All stabilized patients were re-diagnosed 6 months after the initial examination, and the diagnosis was stable in all cases.	Audiovisual integration/detection task. Instructions: “look and listen”.	1) Simulation - simultaneous presentation of a visual stimulus (flickering checkerboard) and auditory stimulus (drumbeats) 2) Fixation	fMRI	“General linear model, 2-stage analysis procedure contrast. Data were analyzed by modelling the different conditions as boxcar functions convoluted with the hemodynamic response function. The contrast was +1 for AV stimulation and –1 for the fixation condition. Data were analyzed by including the contrast images of all subjects of each group into a second-level group analysis. One-sample *t-*tests were performed to test for in-group correspondences and 2-sample *t-*tests to identify areas that are less active in SP compared with HC.”	“The study supports the concept of widespread cortical and subcortical deficits in schizophrenia. The findings suggest abnormal functioning early in the information processing and in high-order association cortices already at illness onset, before the administration of medication or the most confounding effects of illness duration. The main regions have been implicated in visual motion perception and discrimination as well as in attention to sensorial events and perceptual synthesis.”
Hanlon et al. (2016)	33; 6/27; -; 38.0 (13.66); (12.61 ± 1.77 years of education). Does not say how many participants were diagnosed with SP or SP-AFF.	34; 6/28; -; 37.50 (13.35); (12.74 ± 1.83 years of education)	DSM-IV; SCID; PANSS 58.67 (13.71); WTAR- 55.08 (12.96); Calgary Depression Scale 3.30 (3.87); FTNT 0.55(1.00); Olanzapine equivalent 11.77 (7.48); SAS 1.30 (1.59); AIMS 1.18 (2.16); BAS 0.24 (0.50); UPSA	WTAR 58.14 (7.36) as an estimate of pre-morbid intelligence	Age, gender, education, no history of neurological disorder, head trauma with loss of consciousness greater than 5 min, no active substance dependence or abuse (except for nicotine) within the past year, no lifetime history of dependence or use within the last 12 months of hallucinogens, amphetamines or cocaine, or intellectual disability. All abstained from smoking and caffeine intake for at least 1 h before. For HC: no history of a psychotic disorder in a first-degree relative, a current or past psychiatric disorder (with the exception of one lifetime depressive episode), depression or antidepressant use within the last 6 months, or lifetime antidepressant use of more than 1 year. For SP: all patients were taking stable doses of antipsychotic medications. All were drug tested.	Audiovisual detection task Instructions: Participants pressed a button with their right index finger to indicate target detection	1) visual blue box and auditory tone presented simultaneously (2000 Hz). 2) White fixation cross on black background and quiet	fMRI	Group × Time voxel-wise linear mixed-effect ANCOVA was used to examine group differences. Time: 0 to 20s post-stimulus onset.	“During a simple multisensory detection task, SP exhibited abnormal PSU after normal task-related activation in secondary unisensory areas, as well as abnormal task-induced deactivation in the aDMN, and the two hemodynamic abnormalities were related. Current results are consistent with the view that separate neural processes underlie the two phases of the HRF and that they are differentially affected in SP.”
Liu et al. (2016)	17; 1/16; -; 16.9 (9.66)	18; 1/17; -; 44.6 (7.94)	SCID, Axis I and II. WAIS full scale; 92.1 (11.15) PANSS; 80.50 (30.79). Onset age, 26.2 (9.25) Illness duration, 16.7 (9.19) Antipsychotic medication, 17	SCID, Axis I and II. WAIS full scale; 102 (14.40)	Age, gender, handedness, normal hearing, normal/corrected vision, no history of neurological illness, no history of DSM-IV diagnosis of drug or alcohol abuse, parental socioeconomic status, verbal IQ above 75, no hearing, vision or upper body impairment. For HC no history of psychiatric disorder in oneself or first-degree relatives.	Face-voice discrimination task Instructions: Press a response button to a monkey face, press a button to a monkey voice or press a button to a monkey face-voice	1) Visual stimuli of 36 human and 3 primate neutral faces. 2) Auditory stimuli of 36 neutral human sounds and 3 primate digitized voices. 3) Audiovisual stimuli of 36 congruent human face-voice stimuli and 9 primate combinations.	EEG; ERP	“The amplitude and latency of parieto-occipital P100, N170 and P270 were separately submitted MANOVA with group as between-subjects factor, and condition and electrode as within-subjects factors. The amplitude and latency of fronto–central N100 and P200 were separately submitted to MANOVA with group as between-subjects factor, and condition, region as within-subjects factors. To assess the differences in multisensory integration between groups the difference ERP waveforms were obtained where the ‘sum’ ERPs were subtracted from the ‘multimodal’ ERPs for each group. Then, between-group comparisons of the difference ERP waves were conducted with time point-by-time point independent samples *t*-tests.”	“There were no group differences in the multisensory effects as tested with the time point analysis. Differences emerged in the traditional ERP analyses: while the N250 was more negative in the audiovisual relative to the visual condition in both groups, the P400 was reduced to faces but not to face–voice information in the schizophrenia group. In group comparisons, the face processing difficulty existed both to face alone and face–voice stimuli and extended to a lack of facilitatory effects of face–voice stimuli on P270 latency in the schizophrenia group.”
Müller et al. (2014)	15; 4/11; -; 35.1 (9.26)	15; 3/12; -; 40.8 (10.67)	SCID, BRPS; 46.93 (9.68), SAPS; 29.80 (21.6), SANS; 37.80 (13.3), Illness duration, 14.33 (9.12) Antipsychotic medication, 12		Age, gender, education, handedness, normal or corrected-normal vision, tested negative on a urine drug screening. For HC: no history of neurological or psychiatric disorder, including substance abuse, or any history of an Axis I disorder in a first-degree relative. For SP: No comorbid psychiatric or neurological illness, substance abuse or addiction in the last 6 months.	Audiovisual emotion discrimination task. Instructions: Participants were instructed to ignore the sound and blurred face and to just rate the expression of the clear face. The task was to respond as fast and accurately as possible as soon as the eight-point rating scale was displayed. The rating scale, ranging from extremely fearful to extremely happy, was visualized by eight buttons on the screen.	Visual stimuli (V): a face demonstrating happy (H), fear (F) or neutral (N). Auditory stimuli (A): a voice demonstrating happy (H), fear (F) and neutral (N) 1) HV + HA 2) HV + FA 3) HV + NA 4) FV + HA 5) FV + FA 6) FV + NA 7) NV + HA 8) NV + FA 9) NV + NA	EEG; ERP	“ERP sounds: MANOVA with the independent variables emotional expression of the sound and group and the dependent variables amplitudes of P1, N1 and P2 at Cz was calculated to test for group differences. ERPs visual: MANOVA with the independent variables facial emotional expression, side, caudality and group and the dependent variables amplitudes of P1, N1, and P2 was calculated. ERPs MSI: ANOVAs were conducted for the dependent variables P1 and P2 amplitudes, respectively, and the independent variables facial emotional expression, side, caudality, auditory congruency and group.”	“In summary, this study demonstrates deficits in early visual face processing in schizophrenia, which may be counteracted or attenuated by concurrently presented emotionally congruent sounds.”
Roa Romero et al. (2016a)	17; 5/12; -; 35.24 (7.73)	17; 4/13; -; 36.00 (8.29)	DSM-IV and ICD-10, PANSS; 72.41 (5.80), BACS; 245.65 (43.31), Illness duration 8.24, (4.47) Antipsychotic medication, 17	SCID, BACS; 273.47 (37.37)	Age, gender, education, handedness, normal hearing and normal or corrected to normal vision, no record of neurological disorder, no alcohol or substance abuse, 45% underwent multidrug-screening test.	Crossmodal prediction error processing, discrimination task. Instructions: The participants' tasks were to respond to the occasional auditory syllable /fa/ and to an occasional change of the fixation cross, which turned into a white circle.	1) High predictive and congruent (e.g., /Pa/ + /Pa/) 2) high-predictive and incongruent (e.g., /Pa/ + /La/) 3) low-predictive and congruent (e.g., /Ga/ + /Ga/) 4) low-predictive and incongruent (e.g., /Ga/ + /Ta/)	EEG; ERP and oscillatory activity	“Analysis of event-related activity focused on global field power (GFP). The analysis of oscillatory responses focused on oscillatory power to high- and low-predictive congruent and incongruent trials. We focused the analysis on group differences with respect to the PE, which was expressed as the difference between neural responses to incongruent vs. congruent trials. The differences between congruent and incongruent syllables were entered into running repeated-measures ANOVAs with the factors Group and Predictability. Running ANOVAs for GFP data were computed for each sample point in a 0 to 500 ms interval following auditory syllable onset. Cortical sources of main effects or interactions in the analysis were visualized by a local autoregressive average (LAURA) analysis. Oscillatory power was analyzed for a region of interest (ROI) encompassing 10 fronto-central electrodes. Running ANOVAs with the same factors as in the GFP analysis were computed for each sample point and frequency in a 0 to 1000 ms interval following auditory syllable onset”	“The study demonstrates a crossmodal PE processing deficit in SP. In auditory areas we observed similar processing of audiovisual stimulus incongruence in SP and HC. The key novel finding is that SP lack a crossmodal PE processing-related enhancement of frontal theta-band oscillations. The reduced frontal theta-band power presumably reflects a top-down crossmodal processing deficit”.
Roa Romero et al. (2016b)	21; 4/10; -; 35.57(6.55)	21; 4/10; -; 36.79 (7.18)	DSM-IV-TR, ICD-10, PANSS; 74.50 (7.43), BACS; 248.71 (43.76). Illness duration, 9.50 (5.63) Antipsychotic medication, 14	SCID non-patient edition, BACS; 266.50 (39.70)	Age, gender, education, handedness, normal hearing, normal or corrected vision, no neurological disorder, alcohol or substance abuse. Multi drug screening; none positive (45% of participants). For HC: No psychiatric disorder. For SP: No other psychiatric disorder.	McGurk Illusion Instructions: Indicate by button press with the index, middle, ring or small finger of their right hand whether they had perceived the syllable /pa/, /ga/, /ka/, “or something else” respectively	1) Congruent syllables (/pa/, /pa/). 2) Incongruent syllables (/pa/, /ka/). 3) McGurk illusory syllables (/ga/, /pa/)	EEG; ERP and oscillatory activity	“The analysis of EEG data focused on the comparison of ERPs and oscillatory responses to McGurk illusion and congruent trials. Examined ERP amplitudes and oscillatory power at a medio-central region of interest (ROI), comprising 16 channels. For ERPs, GFP, as well as oscillatory power running 2 × 2 ANOVAs with the factors Group and Condition were conducted for each sample point. ERPs were analyzed in a time window from 0 to 500 ms and oscillatory responses from 0 to 850 ms following auditory syllable onset. Significant main effects or interactions were followed-up by *t*-tests”.	“Taken together, our study revealed altered early and late processing of McGurk illusion trials in ScZ. The early ERP effect might reflect audiovisual processing deficits in ScZ patients. The altered late alpha-band suppression effects could reflect abnormal multisensory integration in auditory and/or frontal areas. Our study provides new insight into the processing of the McGurk illusion in ScZ and fosters the notion that alpha-band oscillations reflect altered multisensory integration in ScZ patients”.
Sanfratello et al. (2018)	53; 9/44; -; 39.5 (13.6)	56; 17/39; -; 36.3 (12.0)	SCID-IP, PANSS Positive 14.8 (4.9), PANSS Negative 14.9 (5.1), UPSA, IQ; 101.2 (21)	SCID-NP, IQ; 116.5 (13)	Age, gender, education, no history of neurological disorder, significant head trauma, no current diagnosis of substance abuse. For HC: no history of psychiatric disorder and no first-degree relatives with a history of a psychotic disorder.	Audiovisual discrimination task Instructions: Participants were asked to indicate whether the stimulus was near to them or far from them by pressing one of two buttons on a response device with either their right index finger or right middle finger, respectively	1) Near visual stimulus. 2) Far visual stimulus. 3) Near auditory stimulus. 4) Far auditory stimulus. 5) Near audiovisual synchronous stimulus. 6) Far audiovisual synchronous stimulus. 7) Near audiovisual asynchronous stimulus. 8) Far audiovisual asynchronous stimuli	MEG; ERP	“The ROIs investigated were bilateral: occipital pole, insula, STS, STG, SPG, IPG, and IPS. A 3-way mixed ANOVA was conducted, for both amplitude of activation and for peak amplitude latency, with group entered as a between-subjects factor, and hemisphere, location of stimulus, and presentation of stimuli entered as within-subjects factors. A hierarchical regression analysis was performed with RT as the independent variable; amplitude was entered first, followed by group, and the interaction term last. A second hierarchical regression analysis was performed for latency, with RT as the independent variable; latency was entered first, followed by group, and the interaction term last.”	“Our work here provides evidence of significant brain activation differences in SP vs HC in parietal areas, e.g. areas involved in multisensory integration, particularly IPS. Our results provide additional evidence of a dorsal visual stream impairment in SP.”
Stekelenburg et al. (2013)	18; 1/17; -; 38 (9)	18; 1/17; -; 39 (8.1)	DSM-IV-TR, M.I.N.I, BPRS; 41.4 (11.5). Illness duration, 16.2 (5.6) Antipsychotic medication, 18		Age, gender, education, handedness, normal hearing and normal/corrected vision, no neurological illness, no alcohol or drug dependence or abuse within last year, or long duration of past abuse, no history of electroconvulsive treatment, no medications which would affect EEG.	Audiovisual integration based on temporal and content information. Instructions: No instructions were given	1) Visual only, one of the videos without sound. 2) Auditory only, one of the four sounds against a black background. 3) Audiovisual congruent, video with synchronized original sound. 4) Audiovisual incongruent, auditory /fu/ combined with visual /bi/, auditory /bi/ with visual /fu/, auditory hand clapping with visual tapping of a spoon, and auditory tapping of a spoon with visual hand clapping	EEG; ERP	“N1 and P2 amplitude and latency scores were separately entered in a repeated measures MANOVA with the between-subject factor Group, the within-subject factors Stimtype and Modality. An additional analysis involved the spatiotemporal dynamics of the difference of AV interaction between the two groups by conducting point-by-point two-tailed *t*-tests at each electrode in a 1–300 ms window. In the first analysis the intersensory effect was investigated. We computed the (AVC − [A + V]) difference wave for both groups and performed the running *t*-tests between both difference waves. In the second analysis we examined the AV congruency effect by comparing the (AVC − AVI) difference wave between groups. As a final step, the neural sources underlying audiovisual interactions were estimated by using a linear distributed inverse solution based on a Local Auto-Regressive Average (LAURA) model of the unknown current density in the brain.”	“To conclude, we found clear evidence that patients with schizophrenia show degraded audiovisual integration at the neural level. From a functional perspective, patients with schizophrenia may have difficulties in predicting sound onset and content from vision.”
Stone et al. (2011)	14; 3/11; -; 42.21 (12.69)	15; 5/10; -, 37.80 (13.81)	WASI FSIQ; 98.29 (19.06), PANSS: 54.29 (15.79). Antipsychotic medication, 14	WASI FSIQ; 113.27 (13.47)	Age, gender, education, ethnicity.	Audiovisual discrimination task Instructions: Participants were asked to indicate whether the stimulus was near to them or far from them by pressing one of two buttons on a response device with either their right index finger or right middle finger, respectively	1) Near visual stimulus in the peripheral view. 2) Far visual stimulus in the central view. 3) Near auditory 80 dB sound. 4) Far auditory 64 dB sound. 5) Near multisensory, visual stimuli and auditory stimuli presented 0-5 ms later (appears simultaneously). 6) Far multisensory visual stimuli and auditory stimuli presented 0-5 ms later (appears simultaneously).	EEG; evoked potentials	“EEG evoked potentials in response to visual-only stimuli (VEP), auditory-only stimuli (AEP) and audio-visual stimuli (AVEP) were compared in SPs and HNVs. Maps were generated every 15 ms from 50 to 200 ms post-stimulus. SP and HNV maps were then visually compared at each 15 ms time point. The electrodes and time points detected by *t*-tests delineated spatio-temporal regions where significant group differences in amplitudes occurred. In the present study, the sum of VEP and AEP amplitudes (SUM) was subtracted from the AVEP amplitude at each electrode and time point [AVEP − (AEP + VEP)]. This measure was calculated for each participant separately and group averaged. Between-group comparisons of this measure were assessed with time point-by-time point independent samples *t*-tests. MANOVAs were used to test specific interactions and directions of any effects observed in the *t*-tests.”	“Schizophrenia patients displayed increased physiological multisensory facilitation compared to the control group despite significant unisensory deficits. Our results indicate that basic multisensory processing, at least under some conditions, may represent one type of spared functioning.”
Stone et al. (2014)	46; 7/39; -; 39.2 (13.9)	57; 17/40; -; 39.4 (12.7)	DSM-IV, WASI; 101.5 (17.1)	SCID NP, WASI; 119.9 (11.3)	Age, gender, no neurological disorders or prior head trauma, no substance abuse. For HC: history of psychiatric disorder in oneself or their first-degree relatives.	Audiovisual discrimination task Instructions: Participants were asked to indicate whether the stimulus was near to them or far from them by pressing one of two buttons on a response device with either their right index finger or right middle finger, respectively	1) Near visual stimulus. 2) Far visual stimulus. 3) Near auditory stimulus. 4) Far auditory stimulus. 5) Near audiovisual synchronous stimulus. 6) Far audiovisual synchronous stimulus. 7) Near audiovisual asynchronous stimulus. 8) Far audiovisual asynchronous stimuli	MEG; oscillatory activity	“Event-related oscillations were determined by identifying significant increases or decreases in baseline-corrected spectral power within subject group in the 0–480 ms time window. Significance was determined by one-sample *t*-tests. No group differences were found which did not overlap with event-related oscillations showing either significant increases or decreases in power relative to baseline. Spectral power was compared between HC and SP across the 30–50 Hz (gamma-band) frequency range from 0 to 480 ms post-stimulus for each condition. Independent sample *t*-tests comparing SP to HC were applied at each sensor, time, and frequency point. The results of these *t*-tests were then used to identify candidate clusters of significant group differences in gamma-band power.”	“In summary, this study demonstrates that group differences between SP relative to HC in gamma-band activity are present in response to both unisensory and multisensory stimuli. Yet, the unisensory deficits do not directly map onto changes in gamma-band power in response to multisensory stimuli.”
Straube et al. (2013)	16; 6/10; 19–53; 38 (-).	16; 0/16; 19–47; 27.9 (-)	DSM-IV; PANSS: 64(19); SAPS: 37(27); SANS: 22(27).	–	Handedness, normal hearing, normal vision, German was their primary language. For HC: no medical, neurological or psychiatric illness, past or present. For SP: No additional neurological and medical impairments as well as any cerebral abnormality, stable doses of atypical antipsychotic medication	Implicit audiovisual encoding task Instructions: Participants instructed to watch the videos and respond each time they saw a new picture appear by pressing a button with the left index finger (to ensure attention to task).	Combinations of speech and gestures: 1) Co-verbal gestures; concrete sentence content 2) Co-verbal gestures; abstract sentence content. 3) Sentences without gestures. 4) Gestures without sentences	fMRI	Analysis was performed by entering contrast images into a flexible factorial analysis as implemented in SPM8, in which subjects are treated as random variables. Baseline contrasts was the four conditions. The conjunction of bimodal in contrast to both unimodal conditions was calculated. The interaction between group and co-verbal gesture condition was calculated.	“Our data indicate intact neural integration processes for iconic co-verbal gestures in patients with schizophrenia and specific aberrant activation for metaphoric co-verbal gestures. This indicates intact perceptual-matching processes within the pMTG/STS and dysfunctional higher order relational processes in the left IFG.”
Straube et al. (2014)	16; 6/10; 19–53; 38 (-). Subjects and paradigm is the same as in Straube et al. (2013), just other analysis.	16; 0/16; 19–47; 27.9 (-)	DSM-IV; PANSS: 64(19)	–	Handedness, normal hearing, normal vision, German was their primary language. For HC: no medical, neurological or psychiatric illness, past or present. For SP: No additional neurological and medical impairments as well as any cerebral abnormality, stable doses of atypical antipsychotic medication.	Implicit audiovisual encoding task Instructions: Participants instructed to watch the videos and respond each time they saw a new picture appear by pressing a button with the left index finger (to ensure attention to task).	Combinations of speech and gestures: 1) Co-verbal gestures; concrete sentence content 2) Co-verbal gestures; abstract sentence content. 3) Sentences without gestures. 4) Gestures without sentences	fMRI	A PPI analysis was conducted to assess differences in neural connectivity of the area around the left STS (including parts of the MTG and STG) for groups and conditions. On the group level, a random effects analysis (full factorial analysis) was conducted. Common connectivity patterns across groups and conditions were analyzed and interaction analyzes of group by condition.	“Data indicate a reduced connectivity in patients, between brain regions normally involved in the processing of gestures in an abstract sentence context. It seems that processes that require the connection of brain regions in close proximity are intact in patients, whereas processes relying on the interplay of more distant regions are disturbed most dramatically. Thus, the disconnectivity between STS and IFG might be the basis of dysfunctional integration of metaphoric coverbal gestures and related interpersonal communication problems in patients with schizophrenia.”
Szycik et al. (2009)	15; 8/7; -; 38.2 (10.6)	15; 8/7; -; 36.5 (9.4)	DSM-IV; BPRS 24.1 (8.2).	–	Age, gender, handedness, native speakers of German. For HC: No previous or current psychiatric or neurological disorders. For SP: under antipsychotic treatment (chlorpromazine equivalent in mg/day was 380 ± 222), mean illness duration was 9.9 ± 9.4 years.	Audiovisual speech perception task Instructions: “To keep participants attending to the stimuli, they were required to identify words belonging to a specific semantic target category (i.e. animals) by pressing a button with the left/right index finger depending on whether a target was present/not present.”	1) “Video segments with congruent audiovisual information (lip movements fitting to the spoken word; AV-congruent condition)”. 2) “Video sequences with incongruent information in the audio and video channels (lip movements did not match the spoken word; AV-incongruent condition; e.g., video: insel/island, audio: hotel/hotel).	fMRI	Statistic model: design matrix including all conditions of interest specified using a hemodynamic response function. Multi-subject random effects (RFX). ANOVA, with within-subject factor AV-Con or AV-Incan and between-subject SP and HC.	“Robust differences were found between schizophrenia patients and matched control participants in the processing of audiovisual speech stimuli. The overall pattern of results suggests an abnormality of the patients in right medial and inferior frontal brain regions, possibly related to impaired processing of audiovisual speech by a mirror neuron system.”
Szycik et al. (2013)	15; 8/7; -; 38.2 (10.6). Subjects and paradigm is the same as in Szycik et al. (2009), just other analysis.	15; 8/7; -; 36.5 (9.4)	DSM-IV; BPRS 24.1 (8.2).	–	Age, gender, handedness, native speakers of German. For HC: No previous or current psychiatric or neurological disorders. For SP: under antipsychotic treatment (chlorpromazine equivalent in mg/day was 380 ± 222), mean illness duration was 9.9 ± 9.4 years.	Audiovisual speech perception task Instructions: “To keep participants attending to the stimuli, they were required to identify words belonging to a specific semantic target category (i.e. animals) by pressing a button with the left/right index finger depending on whether a target was present/not present.”	1) “Video segments with congruent audiovisual information (lip movements fitting to the spoken word; AV-congruent condition)”. 2) “Video sequences with incongruent information in the audio and video channels (lip movements did not match the spoken word; AV-incongruent condition; e.g., video: insel/island, audio: hotel/hotel).	fMRI	Functional connectivity analysis with SPM8. General Linear Model. Three seeds were selected a priori: the left IFG, right IFG and right posterior superior temporal sulcus (RpSTS). One-sample *t*-test for each group in each condition for the left IFG seed (Broca's area). ANOVA with group and stimulus.	“Differences in functional connectivity in particular of Broca's area during the processing of AV speech were found between patients with schizophrenia and HC, in particular for incongruent stimuli. Whereas control participants showed a reduced connectivity of Broca's area to other regions in this condition, SP showed increased connectivity which, we propose, is maladaptive in this case as AV integration is futile.”
Wroblewski et al., 2020	17; 4/13; -; 33.12 (12.35); education years: 11.82 ± 1.77	17; 4/13; -; 32.65 (10.07); education years: 12.76 ± 1.39	ICD-10; SAPS 15 (6.89); SANS 9 (6.02); TMT A 31.49 (10.73); TMT B 68.56 (37.8); Digit Span forward 7.94 (1.75); Digit Span backward 6.35 (1.93); Verbal IQ 28.88 (5.25); Proverb Interpretation Task 1.38 (0.46); BAG; Word fluency test WTF	TMT A 26.45 (10.12); TMT B 54.18 (19.44); Digit Span forward 8.06 (2.51); Digit Span backward 6.59 (2.58); Verbal IQ 28.47 (3.91); Proverb Interpretation Task 1.13 (0.20); BAG; Word fluency test WFT	Age, gender, education, handedness, normal or corrected to normal vision, no neurological diseases, no substance use or alcohol abuse within the past six months, brain injury, native German speakers without any knowledge of Russian language. For SP: All except one received antipsychotic treatment; six were additionally treated with antidepressive or other psychiatric medication.	Content judgement task Instructions: Participants were instructed to decide (via button press with the index or middle finger of the left hand) whether the presented stimulus was rather object- or person-related. Participants were instructed to respond as soon as they decided on an answer.	1) An intrinsically meaningful gesture in context of a German sentence. 2) An intrinsically meaningful gesture in context of a Russian sentence 3) A German sentence with emblematic or tool-related content without being accompanied by any gesture	fMRI	Defined three experimental contrasts of interest. Verbal effect, gestures effect and conjunction null (between both unimodal conditions). First-level design matrices were specified, containing an “integration regressor”, a “verbal regressor” and a “gestural regressor”. Dynamic Causal Modelling to analyze effective connectivity, one-sample *t*-tests, Kolmogorov-Smirnov test. Comparison between groups we used one-way ANOVAs and *t*-tests (unpaired, two-samples, one-tailed, Bonferroni-corrected), correctional analyses, within-subject flexible factorial analysis.	“Across all groups, fMRI analyses revealed similar activation patterns […] However, patients revealed significantly reduced connectivity in the verbal pathway (from left middle temporal gyrus (MTG) to left STS). Taken together our results demonstrate the relevance of the pSTS as integration site during gesture-speech integration […] and show, that these processes are generally intact in SP. However, we provide evidence that patients might suffer from neural impairments within the verbal pathway during gesture-speech integration, which is related to concretism and SANS subscores.”
Wynn et al. (2014)	33; 10/23; -; 48.4 (9.0)	30; 9/21; -; 48.0 (8.1). Antipsychotic medication, 33	SCID, Axis I. SANS, BPRS; 36.7 (10.0)	SCID-I and SCID-II	Age, gender, IQ over 70, neurological illness or significant head injury with loss of consciousness for greater than 60 min. No substance dependence in the last 6 months, substance abuse in the last month. For HC: no history of psychotic disorder, bipolar disorder, recurrent depression, substance dependence, paranoid, schizotypal, schizoid, personality disorder or history of psychotic disorder among first-degree relatives.	Audiovisual detection task Instructions: Subjects were instructed to push the mouse button as quickly as possible whenever they heard or saw any stimulus.	1) Visual stimulus: a black letter X. 2) Auditory stimulus: 1000 Hz tone. 3) Audiovisual stimulus: X and tone simultaneously	EEG; ERP	“To examine MSI effects on ERPs, we compared the AV condition to the sum of the unisensory conditions (A + V) for the same set of electrodes, within each group. We analyzed each of the four ERP components separately using a 2 (group) × 2 (AV vs. A + V) repeated measures ANOVA. For all repeated measures ANOVAs resulting in more than one degree of freedom we used Greenhouse-Geisser correction factors (ε) to account for the effects of violated sphericity”.	“In conclusion, our results show that schizophrenia patients exhibit relatively intact MSI, though with some subtle (non-significant) differences at the neural and behavioral levels. Our findings are inconsistent with most (but not all) of the previous studies of MSI in schizophrenia that have reported MSI deficits. Patients with schizophrenia clearly have well-established problems in unisensory processing.”
